# Conjugation of Hypericin to Gold Nanoparticles for Enhancement of Photodynamic Therapy in MCF-7 Breast Cancer Cells

**DOI:** 10.3390/pharmaceutics14102212

**Published:** 2022-10-18

**Authors:** Dimakatso Mokoena, Blassan P. George, Heidi Abrahamse

**Affiliations:** Laser Research Centre, Faculty of Health Sciences, University of Johannesburg, P.O. Box 17011, Johannesburg 2028, South Africa

**Keywords:** photodynamic therapy, hypericin, nanoparticle conjugation, physical adsorption, breast cancer, cell death

## Abstract

Breast cancer, among the different types of cancer, is one of the most diagnosed cancers and the leading cause of mortalities amongst women. Factors, including genetic and epigenetic alterations in tumors, make it resistant to therapies, which results in treatment failures and/or recurrence. Furthermore, the existing therapies have many unfavorable side effects leading to poor prognosis and reduced therapeutic outcomes. Photodynamic therapy (PDT) is one of the most effective cancer therapies with increased selectivity and specificity toward cancer cells. As a result, the use of gold nanoparticles (AuNP) further improves the effectiveness of PDT by increasing the drug loading capacity into the cells. In this study, hypericin (Hyp) photosensitizer (PS) was adsorbed on gold nanoparticles (AuNPs) by sonication to achieve physical adsorption of the PS to AuNP. The resulting compound was characterized by FTIR, Zeta potential, UV-Vis spectroscopy, and TEM. The compound was used for the PDT treatment of MCF-7 human breast cancer *in vitro*. Cellular responses at 12 h post-PDT at 10 J/cm^2^ were observed. Cellular morphology, LDH membrane integrity, ATP luminescence assay, and Annexin V/PI staining were performed. The results demonstrated typical cell death morphological features while the biochemical responses indicated increased LDH and decreased ATP levels. In conclusion, this study presents an insight into the application of advanced PDT in breast cancer cells by inducing cancer cell death *in vitro* via apoptosis.

## 1. Introduction

Breast cancer is the most common cancer in women worldwide. Its prevalence has spiraled over the years due to rapid lifestyle changes, urbanization, and adaptation to Western life. Current treatments include chemo, radiotherapy, and surgery, of which the former two are associated with debilitating side effects such as immunosuppression, cardiovascular disease, diarrhea, hair loss, vomiting, nausea, and many other adverse side effects due to their toxicity to normal cells [1]. Surgery on the other hand is linked to an increased possibility of cancer re-occurrence [1] and disability due to complete or partial loss of the breast depending on the invasiveness of the procedure, which may also affect psychosocial health. In most cases, these conventional cancer therapies are associated with failure, relapse, and an unpleasantly decreased quality of life [2]. Moreover, their high costs always put financial constraints on patients and their families. Therefore, to curb the cytotoxic effects of current cancer treatments, improve the quality of life in cancer patients, and prevent secondary morbidities, a more targeted, safer, and cost-effect treatment modality is required.

Photodynamic therapy (PDT) is a targeted therapy that is minimally invasive and specific to cancer cells by using a selective compound called a photosensitizer (PS), and light within the excitation range of the PS, preferably in the red or near infrared region for tissue penetration. The reaction results in the activation of the PS to a volatile compound in its excited state that reacts with either ground state oxygen in the tumor tissue to produce excited singlet state oxygen or reduces biomolecules in the cells to produce free radicals and other reactive oxygen species (ROS) [3]. It is one of the rapidly evolving forms of therapies globally and is widely used for treatment of several diseases such as bacterial infections, superficial cancers, and skin treatments. As an emerging form of therapy, PDT is in the present day actively being studied and progressive advancements to increase its therapeutic potential are emerging at a fast rate. Important efficacy-enhancing advancements in the current era include the incorporation of nanomaterials as drug carrier molecules. The use of carrier molecules has shifted the attention from the development of new PSs to enhancing the already existing PSs using these nanomaterials. Hence, the use of nanoparticles as PS carrier molecules has been a game changer in PDT and has marked a significant improvement in therapy, as it has curbed the limitations of most PSs, such as poor solubility and reduced cellular uptake [4,5].

Metal nanoparticles (NPs) are the most widely used nanomaterials due to many factors including their ease of synthesis, cost effectiveness, and biological tolerance [6]. Of the different metals, gold, (Au) NPs are one of the most studied due to their widespread availability and unique properties. AuNPs have previously been used as carrier molecules for several PSs and are observed to have an increased potential to cross the endothelial membranes of cancer cells, which is an advantage in PDT since the goal is often to achieve sufficient PS uptake for successful ROS production upon light irradiation [5,6,7,8,9]. Some PSs, with good photosensitization properties have limited ability to move across cell membranes due to their hydrophobicity. Such PSs require the use of a carrier molecule to achieve sufficient and successful uptake and localization of the PS in order that they are used appropriately in PDT. Hypericin is an example of such a PS, and hypothetically, conjugating hypericin to AuNPs will not only enhance cellular uptake to increase drug load into the cells, but also ensure its bioavailability and stability in vivo [10,11]. Therefore, this study sought to enhance the cellular uptake of hypericin in breast cancer cells by using AuNPs as carrier molecules. In the presence of many conjugation methods, physical adsorption was used in order to preserve the structure of hypericin to retain photodynamic characteristics.

## 2. Materials and Methods

### 2.1. Sample Preparation

The hypericin used in this study was purchased from Sigma Aldrich, Johannesburg, South Africa (Catalogue: 1MG-56690). A working stock solution of 2 mM in 1 mL was prepared using dimethyl sulfoxide (DMSO) in the final solution, and stored at 4 °C, covered in foil for protection from direct light. AuNPs were purchased from Sigma Aldrich (Catalogue: 1ML-765457), the particles were 10 nm in size and functionalized with PEG 3000 containing a carboxylic acid terminal. The stock concentration received was used directly at volumes specified below, for each respective experiment. All experiments were conducted under strict aspectic conditions and, where necessary, 70% ethanol was sprayed at regular intervals.

### 2.2. Conjugation of Hypericin to Gold Nanoparticles

Hypericin was physically adsorbed onto AuNPs (Sigma-Aldrich, 1 ML-) [12] by sonication at room temperature for 2 h using the Branson ultrasonic sonicator (Branson MH 2800 Ultrasonic Cleaner, Johannesburg, South Africa). The PEGylated AuNP (carboxyl-PEG 3000) was diluted in distilled water (1:10) to a final volume of 1 mL and placed in a 2 mL microcentrifuge tube. The tube was placed in the sonicator, held midway away from the edges using a retort stand with a clamp holding the tube at the upper end. Then, 20 µL of 2 mM hypericin was added to the AuNP solution continuously at 30 min intervals to a final volume of 100 µL for 2 h.

### 2.3. Chemical Characterization and Localization of the Compound

#### 2.3.1. Optical Properties Using UV-Vis Spectrophotometry

To determine the wavelengths of maximum absorption, the optical properties of the PS, AuNPs, and the compound were investigated by scanning the electromagnetic spectra of all compounds using UV-Vis Spectrophotometry from 200–800 nm at 2 nm wavelength intervals. Samples were each diluted from their stock concentrations to readable concentrations. The obtained spectra were plotted onto line graphs using Origin software. The compound spectral analysis was compared with both the PS alone and AuNP alone before conjugation to check for any changes in characteristics. The concentration of each sample was estimated using the known hypericin concentration and the peak absorbance at 560 nm. The concentration of the PS onto the AuNP was determined by measuring the absorbance of the conjugated material at 560 nm and using the equation: Concentration Test (cT) = (Absorbance of Standard)/(Absorbance of Test) × Concentration of standard.

#### 2.3.2. Assessment of Chemical Structure Using Fourier Transform Infrared Spectroscopy

Fourier transform infrared (FTIR) spectroscopy was performed using the potassium bromide (KBr) pellet method, in which 100 µL of 2 mM hypericin, 100 µL of AuNP and 100 µL of the compound were frozen at −80 °C overnight and lyophilized for analysis. After lyophilization, the samples were thoroughly ground and mixed with dry KBr using a mortar and pestle to form a homogeneous powder. Each sample was placed in a pellet die and pressed at 5000–10,000 psi. Each pellet was carefully removed from the template and placed on the FTIR sample holder (Perkin Elmer, Spectrum 100 FTIR spectrometer, University of Johannesburg, Analytical Chemistry Department) for analysis. The sample pellet had to be nearly clear for accurate result analysis.

#### 2.3.3. Zeta Potential and Size Confirmation

Zeta potential (ZP) and dynamic light scattering (DLS) were performed using the Malvern Zeta Sizer Nano series. ZP was used to determine the surface charges of the individual compounds and the compound. The compounds were diluted in deionized water and homogenized by sonication. DLS and ZP were measured together using the same sample load following protocol described by Crouse et al. 2020 [13]. ZP potential provides information about the stability of the compound in which an increased ZP value denotes good and better colloidal stability [14]. DLS on the other hand measures the size of the particles in a colloidal system [14].

#### 2.3.4. Determination of Particle Size and Structure Using Electron Microscopy

High resolution electron microscopy (HR-EM) was used to determine the size of the particles and their dispersion after conjugation. To measure the size of the particles, a JEM-2100 HR-Electron Microscope (JEOL Ltd., Tokyo, Japan.) was used at varying magnifications. Carbon coated 200 Mesh Cu TEM grids (Lot# 1261229, SPI Supplies) were used as received. AuNP solution and the compound were loaded separately onto the grids by dropwise pipetting, covering the entire surface of the grid, which were then left to airdry overnight in the dark before microscopy. The Cu grids were loaded on the TEM specimen holder and analyzed accordingly. Images were captured and analyzed using ImageJ software (National Institutes of Health and Laboratory for Optical and Computational Instrumentation (LOCI), University of Wisconsin, United States of America). Alongside size determination, the compounds were also analyzed for element presence and intensity using a special feature on the JEM-2100 HR-EM that detects single atoms and their intensities, semi-quantitatively. The graphs generated by the instruments were captured and used without modification.

#### 2.3.5. Subcellular Localization of the Compound

To determine the subcellular localization of the Hyp-AuNP compound, commercially purchased MCF-7 cells (ATCC^®^ HTB-22™) were seeded at a concentration of 1 × 10^5^ in 3.4 cm diameter culture dishes with sterile glass coverslips in place. The cells were cultured in prewarmed complete medium comprising of Dulbecco’s Modified Eagle’s Medium (DMEM), 1% Pen-Strep, 1% amphotericin B, and 5% fetal bovine serum (FBS) and attached on coverslips for 4 h, following incubation cells were washed 2 times with pre-warmed Hank’s balanced salt solution (HBSS). Cells were then treated with the Hyp-AuNP compound at a concentration of 3.8 µM in 3 mL of complete pre-warmed culture media and incubated for 8, 12, and 24 h at 37 °C, 85% humidity, and 5% CO_2_. Following incubation, cells were washed 3 times with pre-warmed HBSS to remove excess PS. Direct staining with 100 nM mitotracker (Invitrogen M7514, ThermoFisher, Johannesburg, South Africa) for mitochondria, 65 nM lysotracker (Invitrogen L7526) for lysosomes, and 65 nM ER tracker for endoplasmic reticulum was done after fixation using 4% paraformaldehyde (Sigma-Aldrich) for 15 min at room temperature. Cells were then washed 3 times with wash buffer followed by permeabilization with 0.5% Triton X-100 in 1X PBS, for 15 min at room temperature. The washing step was repeated followed by the nucleus counterstaining with 300 nM 4,6-diamidino-2-phenylindole, DAPI (Invitrogen, D1306) in 1X PBS for 2 min. Excess stain was washed off with 1X PBS 3 times and the coverslip inverted onto the glass slide with a drop of mounting medium. Slides were then viewed under the fluorescence microscope. Fluorescence images were captured on a Carl Zeiss Axio Z1 fluorescence microscope and were further analyzed using the Zen Pro (3.1) Carl Zeiss software. A separate set of MCF-7 cells was also treated with the same concentration of hypericin alone (not adsorbed to AuNP) at 8, 12, and 24 h in the same culture environment and observed using the same fluorescent microscope.

#### 2.3.6. Phototoxicity of the Compound

MCF-7 cells were seeded at 3 × 10^5^ concentration in 3.4 cm diameter cell culture dishes containing 3 mL of complete culture medium and incubated at 37 °C, with 5% CO_2_ and 85% humidity, to attach. Treatment was done by adding different concentrations (3.8, 7.6, and 15.2 µM) of the compound and irradiated with a 594 nm diode laser (Oriel Corporation, National Laser Centre (NLC), South Africa) at 10 J/cm^2^. Untreated cells were used as controls, which included cells treated with laser light only and cells with compound only. Post treatment cellular response was assessed at 12 h post-treatment incubation using bright field microscopy microscope (Wirsam, Olympus CKX41, Johannesburg, South Africa) for morphological assessment, the CellTiter-Glo luminescence assay (Promega, PRG7570, Johannesburg, South Africa), for analysis of cellular proliferation and the CytoTox 96 non-radioactive cytotoxicity assay (Promega, PRG1780) for direct cytotoxicity. Luminescence for cell proliferation and absorbance for cytotoxicity were measured on the VICTOR Nivo multimode microplate reader (Perkin Elmer, Johannesburg, South Africa) following protocols as described by Chizenga et al. 2020 [15].

#### 2.3.7. Annexin V/PI

To determine the type of cell death caused by the Hyp-AuNP compound, the annexin V-fluorescein isothiocyanate (FITC) apoptosis and necrosis detection kit1 (BD Biosciences, BD Pharmingen™, Johannesburg, South Africa) was used. The percentage of cell populations undergoing apoptosis or necrosis was measured by flow cytometry. MCF-7 cancer cells were cultured and treated with the compound as described previously. From the 3.4 cm diameter plates, cells were added to 5 mL flow cytometric falcon tubes and washed twice with cold PBS. A cell count was done using the trypan blue cell count assay. Cells were then re-suspended in 1X binding buffer at 1 × 10^6^ cells/mL. About 100 µL of re-suspended cells was added to the 5 mL tube and stained with 5 µL of FITC Annexin V and 5 µL of PI. The mixture was gently vortexed and incubated at room temperature in the dark for 15 min. Following incubation, 400 µL of 1X binding buffer was added to each tube, and a flow cytometric analysis was performed using the BD Accuri C6 flow cytometry.

#### 2.3.8. Statistical Analysis

All repeatable experiments were performed at least three times (n = 3) in duplicate and statistical analysis was performed using SigmaPlot software version 14.0. Descriptive statistics was carried out for measures of central tendency (mean, median, and mode), and measures of dispersion (variance, standard deviation, and standard error) for all samples and groups. A Student’s *t*-test was performed to analyze the statistical significance between the control (untreated cells) and experimental groups. Significance is reported as *p* < 0.05 (*), *p* < 0.01 (**), and *p* < 0.001 (***). Standard error is indicated by error bars on bar graphs.

## 3. Results

### 3.1. Characterization

#### 3.1.1. UV-Vis

**Figure 1:**
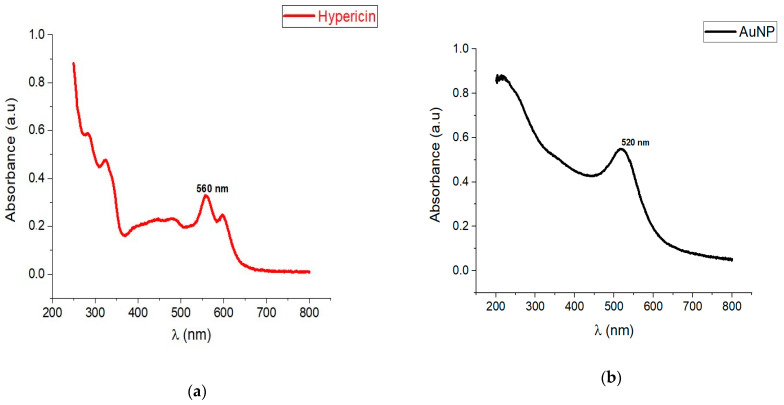

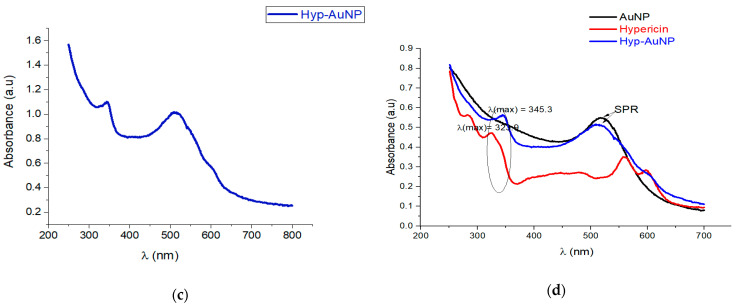
UV-Vis characterization of Hyp-AuNP compound compared to AuNP surface plasmon resonance (SRP) and hypericin peaks (**a**) AuNP spectra, (**b**) hypericin spectra, (**c**) compound spectra, (**d**) mirrored plots.

To confirm the success of the physical adsorption of hypericin on AuNPs, UV-Vis spectroscopy was performed. The optical properties of AuNP alone, hypericin alone, and the Hyp-AuNP compound were studied, and the spectra are shown in Figure 1. The AuNP UV-Vis results showed a surface plasmon resonance (SPR) peak at around 520 nm within the same region as the compound. The hypericin peak was detected at ~340 nm with the same observed for the compound. The compound possessed the same peaks as hypericin and the AuNP thus indicating that hypericin was physically adsorbed to the AuNP. 

#### 3.1.2. FTIR

**Figure 2:**
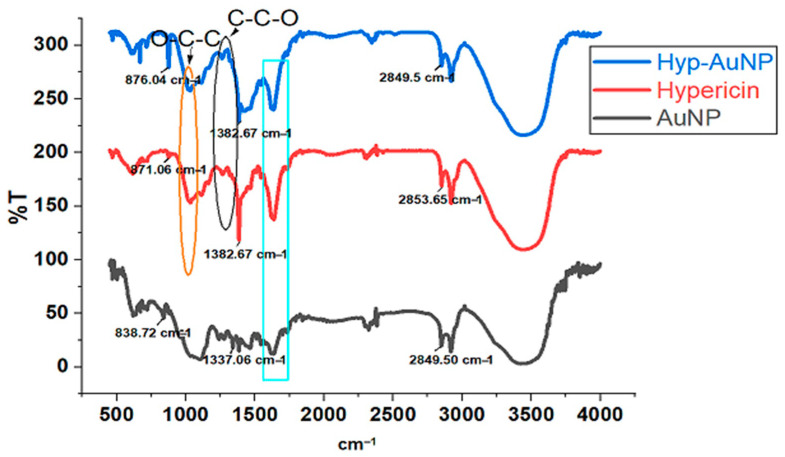
FTIR results of Hyp-AuNP compound, AuNP, and hypericin.

Gold nanoparticle surfaces have affinity to atoms such as S, N, and O while hypericin consists of O atoms which facilitate the physical adsorption on the AuNPs surface. The FTIR results in Figure 2 below show possible O–C–C and C–C–O bonding peaks appearing on both the hypericin and the compound between 1000 and 1300 cm^−1^. Similarly, the hypericin carbonyl peak at 1750 was also observed on the compound. There is a broad –OH stretch at 3500 cm^−1^ on the compound indicating the presence of both AuNP and hypericin which correlates with the structure of hypericin which consists of a lot of –OH bonds. There are also CH bonds Between 2840 and 2950 cm^−1^.

#### 3.1.3. Zeta Potential and DLS

Dynamic light scattering (DLS) was used to measure the size of the Hyp-AuNP compound compared to the AuNP alone. A compound shift and polydispersity index (PdI) was observed, as shown in Table 1, as hypericin increases the hydrodynamic size of the compound. Zeta potential was used to measure the surface charges of AuNP alone, hypericin alone, and the Hyp-AuNP compound. A shift was observed for the Hyp-AuNP compound compared to AuNP and hypericin alone (Table 1).

#### 3.1.4. Particle Size and Dispersion in Solution

**Figure 3:**
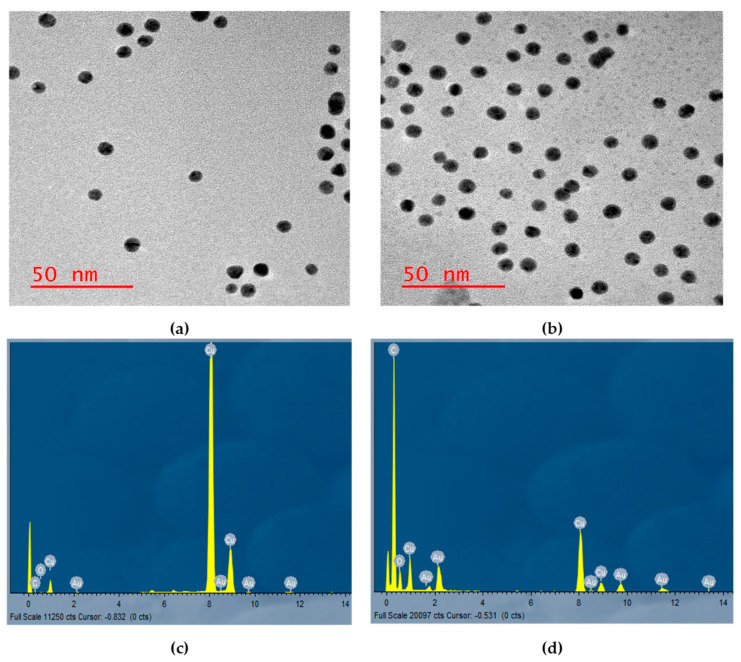
Item Analysis using HR-EM showing the presence and intensity of single elements loaded onto the Cu grids. (**a**) AuNP alone, (**b**) the compound showing an average monodispersion and size averaging 10 nm, (**c**) AuNP alone showing high presence of Au atoms, and (**d**) the compound showing high intensity of C from the C ring of the hypericin molecule and O from the hydroxyl groups.

HR-EM confirmed the size of the AuNP at 10 nm as supplied by the manufacture. The compound did not show marked changes in size by determination of the size using EM however the results confirmed monodispersion of the particles and element was confirmed using atom detection and measurement of intensity. Note that the hypericin particles loaded onto the surface of the TEM may not cause visible alteration of size due to their small size in KDa, hence confirmation of the presence of the molecules using the technique was conducted by observing the element intensity as shown in Figure 3d. Hypericin contains numerous C rings and hydroxyl groups, hence the presence of the element on the grids. H is not captured due to its volatility as a gas that the technique cannot capture as a single element. Figure 3 summarizes the observed findings.

#### 3.1.5. Localization

**Figure 4:**
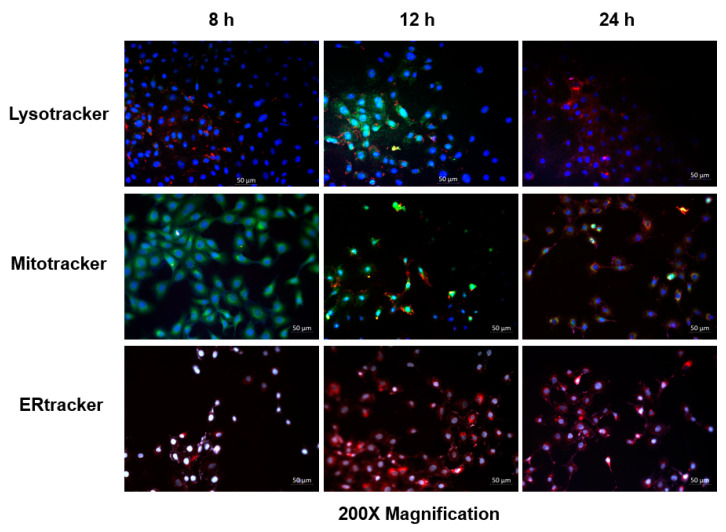
Hyp-AuNP compound localization in MCF-7 cells showing Hyp-AuNP accumulation in the cytoplasm, lysosomes (lysotracker-green) and mitochondrion (mito-tracker-green) at 12 h, indicated by the orange color (mixture of green and red) (Hyp-AuNP). No significant Hyp-AuNP localization on the endoplasmic reticulum (ER tracker) was observed at any incubation time. Cell nuclei were counterstained with DAPI (blue).

**Figure 5:**
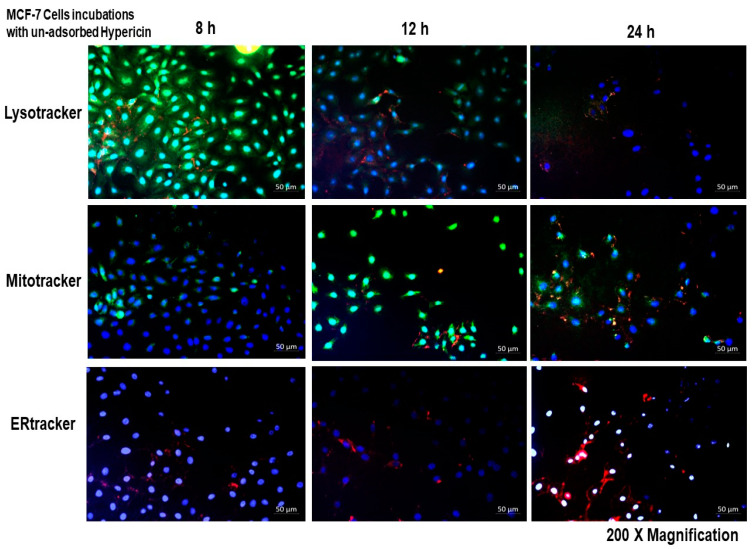
Hypericin localization in MCF-7 cells. A significant amount of hypericin accumulates in the lysosomes and the mitochondrion at 12 and 24 h, indicated by the orange color, mixture of green from the lysotracker and mitotracker and red from hypericin. No hypericin localization on the endoplasmic reticulum (ER tracker) was observed at any incubation time. Cell nuclei were counterstained with DAPI (blue).

The localization of the Hyp-AuNP compound and hypericin alone was checked for autofluorescence in MCF-7 cells and observed under the Carl Zeiss fluorescent microscope using the Zen Pro (3.1) Carl Zeiss software. Significant uptake of the compound was observed at 12 h on MCF-7 cells as shown in Figure 4. This is well illustrated by the combination of the green color of the lysotracker and the red color of the compound to produce an orange color showing that the compound is localizing in the lysosomes. A significant orange color is also observed in the mito-tracker-stained cells after 12 h, indicating the color combination of green from the mito-tracker and red from the compound. The ER tracker showed no significant color change, after compound incubation, there was no overlap of the ER tracker color (white) with the Hyp-AuNP compound (red) at all incubation times. Figure 5 results show the localization of hypericin alone at the same concentration as that of the compound and the same incubation. The unbound hypericin accumulated in the lysosomes at 12 h, the same as the compound, however the intensity is lower than that of the compound. Similarly, the accumulation of the PS alone in the mitochondrion is significantly lower than that of the compound accumulation in the mitochondrion. Additionally, more of the unbound PS accumulates in the mitochondrion at 24 h as compared to the compound which accumulated in the mitochondrion at 12 h. Supplementary information of each of the localization channels has been provided (Localization supplementary information).

### 3.2. Phototoxicity

#### 3.2.1. Cell Morphology

**Figure 6:**
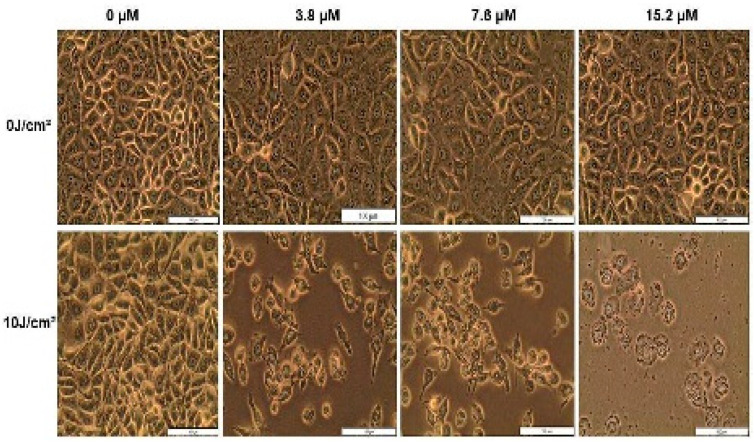
Morphology at 12 h post Hyp-AuNP compound PDT treatment.

MCF-7 cells were treated with the Hyp-AuNP compound and irradiated with a PDT fluence of 10 J/cm^2^. The cells were then incubated for 12 h after PDT. The morphology of PDT treated cells observed using the inverted light microscope (Wirsam Scientific, Olympus CKX41, Johannesburg, South Africa) and the cellSens software showed visible morphological differences compared to the untreated control cells, only laser cells, and only Hyp-AuNP cells. Cells treated with Hyp-AuNP compound PDT began to show visible morphological changes from the lowest concentration to the highest concentration as observed in Figure 6. The observed morphological differences include cell shrinkage, rounding up of cells, floating/detachment from culture plates, and vesicle formation compared to the control cells. These changes indicate cell death due to PDT treatment. All untreated cells, only laser cells, and only Hyp-AuNP treated cells showed no morphological changes, while the laser-only (10 J/cm^2^) cells looked overconfluent and thus very compact compared to the untreated control cells.

#### 3.2.2. LDH

**Figure 7:**
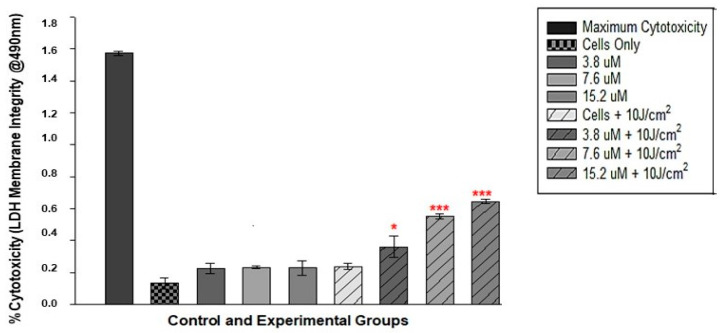
LDH membrane integrity test results after 12 h incubation post PDT irradiation with a laser fluence of 10 J/cm^2^. Significance is shown as * *p* < 0.05, *** *p* < 0.001 (SEM). Experiment was repeated 3 times (n = 3).

LDH release after PDT at 10 J/cm^2^ was observed at different Hyp-AuNP compound concentrations. At 12 h post PDT, statistical significance was observed in all PDT concentrations (3.8 µM + 10 J/cm^2^, 7.6 µM + 10 J/cm^2^ and 15.2 µM + 10 J/cm^2^) as shown in Figure 7. The significance was shown as *p* < 0.05, *p* < 0.01 and *p* < 0.001 respectively in comparison to the control group. There was no statistical significance between the Hyp-AuNP compound only cells and the laser only cells when compared to the control cells.

#### 3.2.3. ATP

**Figure 8:**
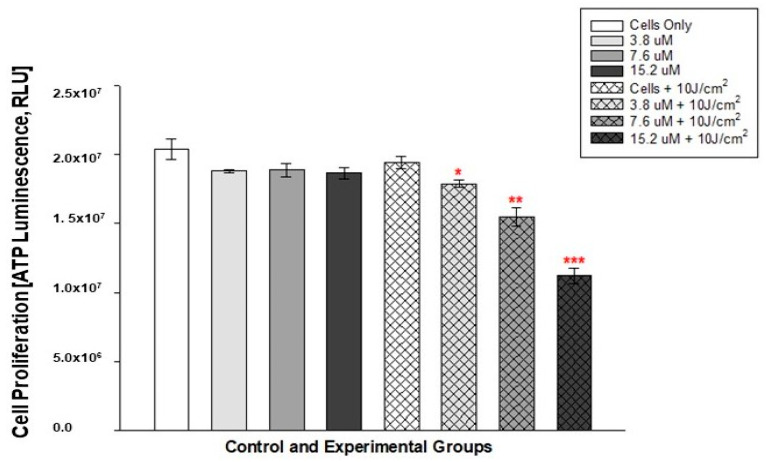
ATP luminescence after 12 h post PDT with a laser fluence of 10 J/cm^2^. Significance is shown as * *p* < 0.05, ** *p* <0.01 and *** *p* < 0.001 (SEM). Experiment was repeated three time (n = 3).

The 12 h and 10 J/cm^2^ ATP results showed statistical significance in all PDT-treated experimental groups (3.8 M + 10 J/cm^2^, 7.6 M + 10 J/cm^2^ and 15.2 M + 10 J/cm^2^). Statistical significance was expressed as *p* < 0.05, *p* < 0.01 and *p* < 0.001, respectively. This then indicates that PDT at a fluence of 10 J/cm^2^ had a significant effect on cells as early as 12 h after irradiation from the lowest to the highest Hyp-AuNP compound concentration. No statistical significance was observed in cells treated with Hyp-AuNP compound and laser alone compared to the untreated control cells, as shown in Figure 8.

#### 3.2.4. Annexin V/PI

**Figure 9:**
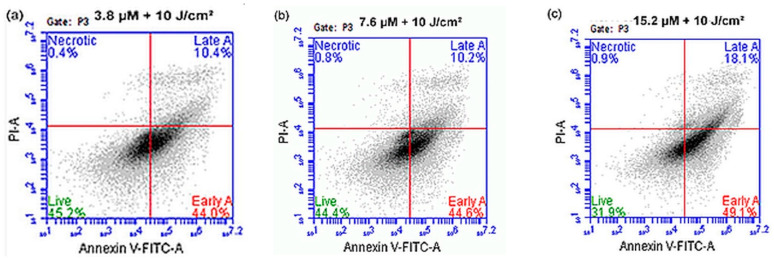

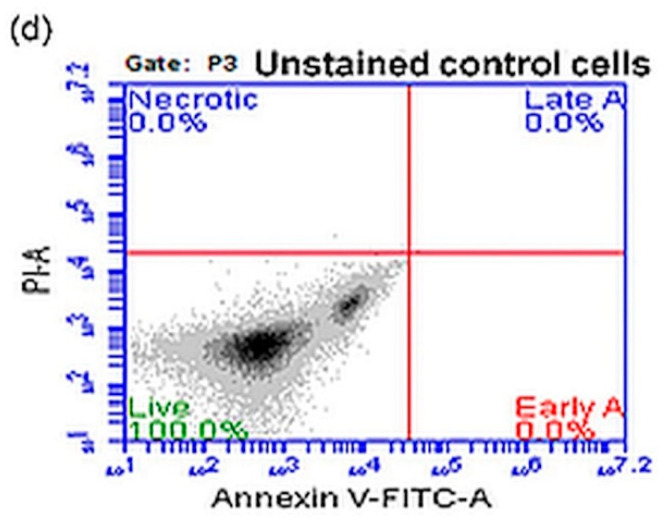
Annexin V/PI flow cytometry results demonstrating that cell death occurs mainly via early and late-stage apoptosis from the lowest to the highest 12 h post-PDT treatment at 10 J/cm^2^. (**a**) 3.8 µM, (**b**) 7.6 µM, and (**c**) 15.2 µM. (**d**) Shows untreated control cells.

Flow cytometric analysis of Annexin V/PI at 12 h post PDT is shown in Figure 9. The results demonstrated that cell death mostly occurred via early and late-stage apoptosis. At 3.8 µM and 10 J/cm^2^ fluence (a), 45.2% of cells were viable while 44% of cells were undergoing early apoptosis, 10.4% late apoptosis, and only 0.2% cells went through necrotic cell death. At 7.6 µM 10 J/cm^2^ (b), 44.4% of cells were viable, while 44.6% were undergoing early apoptosis, 10.2% late apoptosis, and 0.8% were necrotic. At 15.2 µM 10 J/cm^2^ (c), 31.9% of cells were viable, 49.1% were undergoing early apoptosis, 18.1% were going through late apoptosis, and only 0.9% were necrotic.

## 4. Discussion

The ineffectiveness of conventional cancer therapies in breast cancer management and other forms of cancer is a major problem. Since the beginning of modern medicine, many successive discoveries have led to the alleviation of the stress caused by various types of diseases, with much success in most cases. Incomparably, though some progress has been made in oncology, cancer remains problematic as far as treatment is concerned. This is in part due to the nature of the disease, where genetic and epigenetic changes in the tumors, give cancer cells biological features that not only mimic normal cells for immune evasion but also confer resistance to therapies and lead to treatment failure and/or tumor recurrence [16]. In addition, existing therapies are associated with several adverse effects leading to disability, poor prognosis, and relapse. Ultimately, while patients and oncologists have numerous treatment options to choose from, finding the right therapy is always a challenge.

PDT has therefore contributed much to the treatment of some superficial and subcutaneous cancers in an efficient manner. However, due to some limitations as mentioned earlier, enhancement of PDT using available PS-delivery systems is necessary. Hypericin for example is a good PS however with low uptake because of its hydrophobicity. In this study, therefore, hypericin was adsorbed to AuNPs as carrier molecules with the purpose of enhancing cellular uptake with the consequent improved PDT effect on MCF-7 breast cancer cells. Using sonication Hypericin was adsorbed on carboxylic acid functionalized AuNPs with PEG 3000 to form a compound loaded with hypericin molecules. Upon characterization using varying techniques, hypericin was successfully adsorbed onto the AuNPs by observation of the physicochemical characteristics of the compound. UV-Vis spectrophotometry showed the absorption peaks of the individual compounds and the compound. Optical properties were retained in the compounds indicating the functionality of the hypericin molecules while adsorbed on the AuNPs. The advantage of using sonication over chemical alteration for conjugation is that physical adsorption retains the chemical structure of the PS and hence the absorption peaks on hypericin were retained [17,18]. Imaging using HR-EM confirmed the presence of the compound as well as dispersed single molecules in suspension and not as agglomerates. Sometimes sonication may cause agglomeration of compounds especially nonpolar compounds like hypericin, but the methods used in the study, dropwise continuous addition of hypericin in low concentration prevented this from happening. The molecules adsorb slowly onto the AuNPs without cramping each other. HR-EM also confirmed the elements on the compound indicating the presence of both hypericin and AuNPs.

Furthermore, FT-IR analysis confirmed the chemical structure of a nanocompound indicating the presence of intact bonds of hypericin on the compound. Because no new chemical bonds were formed, none could be observed on FT-IR. The final product has a net negative surface charge as observed with ZP analysis. ZP is the average electrostatic charge present on particles suspended in liquids. It provides the difference in electrical charge between solids and liquids in a colloidal system. This information is important for the description of nanoparticle stability, circulation time, particle permeability, and biocompatibility. ZP with a value +/−15 mV indicates instability, and such a solution may need pH adjustment to make the compounds more stable. While the ZP measures the charge of particles in a colloidal system, DLS measures the size of the nanoparticle [14,19,20], DLS confirmed the change in light scattering on the compound. The DLS value was substantially increased indicating the formation of a larger molecule, by conjugation which scatters light semi-proportionally to the average size, and thus showed the higher molecular weight of Hyp, hence confirming the adsorption of hypericin to AuNPs.

Having confirmed the conjugation, the compound was used on MCF-7 cells to check its functionality. MCF-7 cells are harvested from breast adenocarcinoma which is in its in vivo form highly resistant to most chemotherapeutic drugs. These cells were used to check the efficiency of this compound for PDT of breast cancer cells, which among the different types of cancers, is one of the most diagnosed cancers and the leading cause of disability and death in women [21]. Current therapies are less effective and lethal, due in part to the heterogeneity of breast cancer and the presence of different subtypes. PDT has potential to alleviate this problem especially when enhanced with the use of nanoparticles as observed in this investigation. The use of nanoparticles enables a targeted method focused on specific receptors and thereby increases the selectivity of photodynamic therapy. This is achieved through passive or active targeting of the tumor [22]. This compound was shown to enhance cellular absorption of the PS [23]. Such functions include increased in vivo circulation time and increased stability. Because of their biocompatibility, AuNPs can evade recognition and potential interference by the immune system.

Physiologically, the immune system recognizes all foreign substances, including therapeutic drugs, as invaders. When injected intravenously or intramuscularly, PSs may be affected by the host’s immune response. This results in either drug denaturation or other disruption of the drug’s pharmacodynamics in the body. Nanoparticles, including AuNPs, mimic biological components in the body and therefore remain undetected by immune system barriers, creating an excellent delivery system for therapeutic drugs [12]. They can escape opsonization by the reticuloendothelial system but are large enough to be retained in the systemic circulation. Hence, nanotechnology has provided one of the breakthroughs in PDT by using these nanomaterials as carrier molecules that avoid all these limitations in PDT. In this present study, conjugation resulted in improved hypericin cellular uptake as indicated by the subcellular localization study using fluorescence microscopy. In fact, 12 h incubation was enough to get hypericin inside the cells. Interestingly, at 24 h the cells had less hypericin with many possibly attributable causes either technically due to the growth of cells during incubation or actual biological influences, efflux or degradation. These were not further analyzed in the scope of this study and could be a commendable future investigation to ascertain the cause of this observation. At present, inferences made from this observation are that 12 h incubation is sufficient for in vitro localization and over incubation should be avoided.

Post irradiation cellular responses indicated altered morphology, increased cytotoxicity, and reduced proliferation of the cells due to the PDT effect. Increased hypericin-mediated PDT efficacy has been proven by several studies using different enhancement techniques. In a study conducted by Muhleisen and colleagues, they loaded hypericin onto superparamagnetic iron oxide nanoparticles. They observed increased uptake alongside a reduced cellular uptake time of the drug and enhanced PDT effects compared to administration of free-floating hypericin [24]. Similarly, Cheng and colleagues showed that non-covalent conjugation and delivery of a hydrophobic PS ensured efficient release and penetration of the PS into the deep center of the tumor [25]. These observations alongside the finding in this study prove that hypericin conjugation to AuNPs using physical adsorption is a good approach to enhance PDT efficacy in cancer cells. To infer this observation in vivo, the compound is more likely to enhance PDT by increasing the load of Hyp in the tumor tissue because of added advantages resulting from the effects of the enhanced permeability and retention (EPR) effect and other mechanisms like enhancing the flow-through of compounds via transcellular pathways [26]. The increased vasculature and porosity of the blood vessels make it easier for such a compound to enter interstitial spaces in close proximity to the tumor cells, and because of the decreased lymphatic drainage, the compounds are not readily removed from the microenvironment. The increased potential for intracellular absorption of the compound will therefore be both due to the effect of the affinity of cells for the compound and the spatiotemporal allowance that leaves enough time and space for the cells to take up most of the compounds.

## 5. Conclusions

In conclusion, this study demonstrated that using AuNPs for hydrophobic PSs such as hypericin improves the effectiveness of PDT and causes cell death mainly by apoptosis. Due to its reduced invasiveness and selectivity for tumor cells, PDT remains an attractive form of cancer therapy. However, efficient drug delivery to tumor cells is essential and requires full exploration and understanding. In this study, hypericin was physically adsorbed onto AuNPs by sonication, thereby forming a compound with non-covalent bonding. This increased the drug’s accumulation in MCF-7 breast cancer cells and thus increased PDT efficacy from the lowest to the highest concentrations. Therefore, noncovalent Hyp-AuNP conjugation is a promising approach for hydrophobic PS drug delivery to enhance PDT efficacy. Further studies are warranted to demonstrate the effectiveness of PDT and the detailed cell death mechanism, to ensure its application as a treatment option for cancer therapy.

## Figures and Tables

**Table 1:** DLS, PdI, and Zeta potential Results.

Title 1	DLS (d. nm) ^1^	PdI	Zeta Potential
AuNP	23.93	0.10	−14.7
Hypericin	-	0.50	−24.7
Hypericin-AuNP	771.97	0.64	−32.1

^1^ d. nm: diameter values in nanometers; PdI: polydispersity index.

## Data Availability

Not applicable.

## Supplementary Materials

The following supporting information can be downloaded at: https://www.mdpi.com/article/10.3390/pharmaceutics14102212/s1, Localization Supplementary Material.
