# Correction: Mokoena et al. Conjugation of Hypericin to Gold Nanoparticles for Enhancement of Photodynamic Therapy in MCF-7 Breast Cancer Cells. *Pharmaceutics* 2022, *14*, 2212

**DOI:** 10.3390/pharmaceutics17040402

**Published:** 2025-03-24

**Authors:** Dimakatso Mokoena, Blassan P. George, Heidi Abrahamse

**Affiliations:** Laser Research Centre, Faculty of Health Sciences, University of Johannesburg, P.O. Box 17011, Johannesburg 2028, South Africa; drmokoena2@gmail.com (D.M.); blassang@uj.ac.za (B.P.G.)

## Error in Figure

In the original publication [1], there was a mistake in Figure 4 as published. Inadvertently, the wrong image was included for Mitotracker—8 h. The corrected Figure 4 appears below. The authors state that the scientific conclusions are unaffected. This correction has been approved by the Academic Editor. The original publication has also been updated.

## Figures and Tables

**Figure 4:**
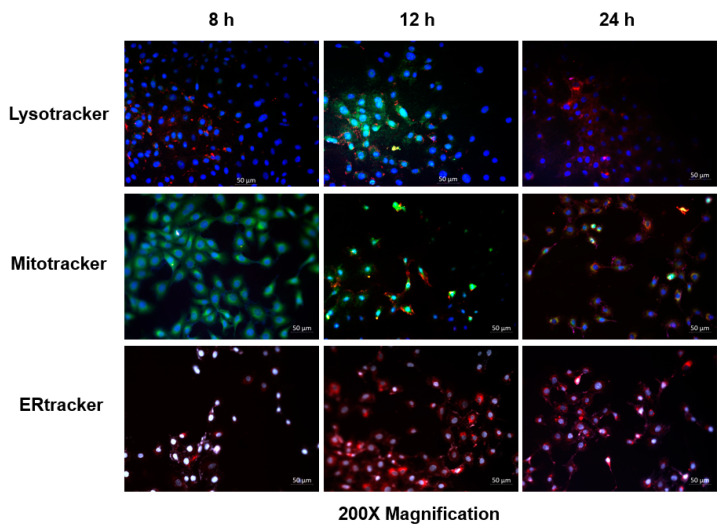
Hyp-AuNP compound localization in MCF-7 cells showing Hyp-AuNP accumulation in the cytoplasm, lysosomes (lysotracker-green) and mitochondrion (mito-tracker-green) at 12 h, indicated by the orange color (mixture of green and red) (Hyp-AuNP). No significant Hyp-AuNP localization on the endoplasmic reticulum (ER tracker) was observed at any incubation time. Cell nuclei were counterstained with DAPI (blue).
