# Supplementary material for: Conjugation of Hypericin to Gold Nanoparticles for Enhancement of Photodynamic Therapy in MCF-7 Breast Cancer Cells
Source: Pharmaceutics. 2022 Oct 18;14(10):2212. doi: 10.3390/pharmaceutics14102212 (PMC9611363; doi:10.3390/pharmaceutics14102212)
Supplement: Supplementary file 1 [file pharmaceutics-14-02212-s001.zip › pharmaceutics-1923110-supplementary.pdf]

LOCALIZATION SUPPLEMENTARY MATERIAL

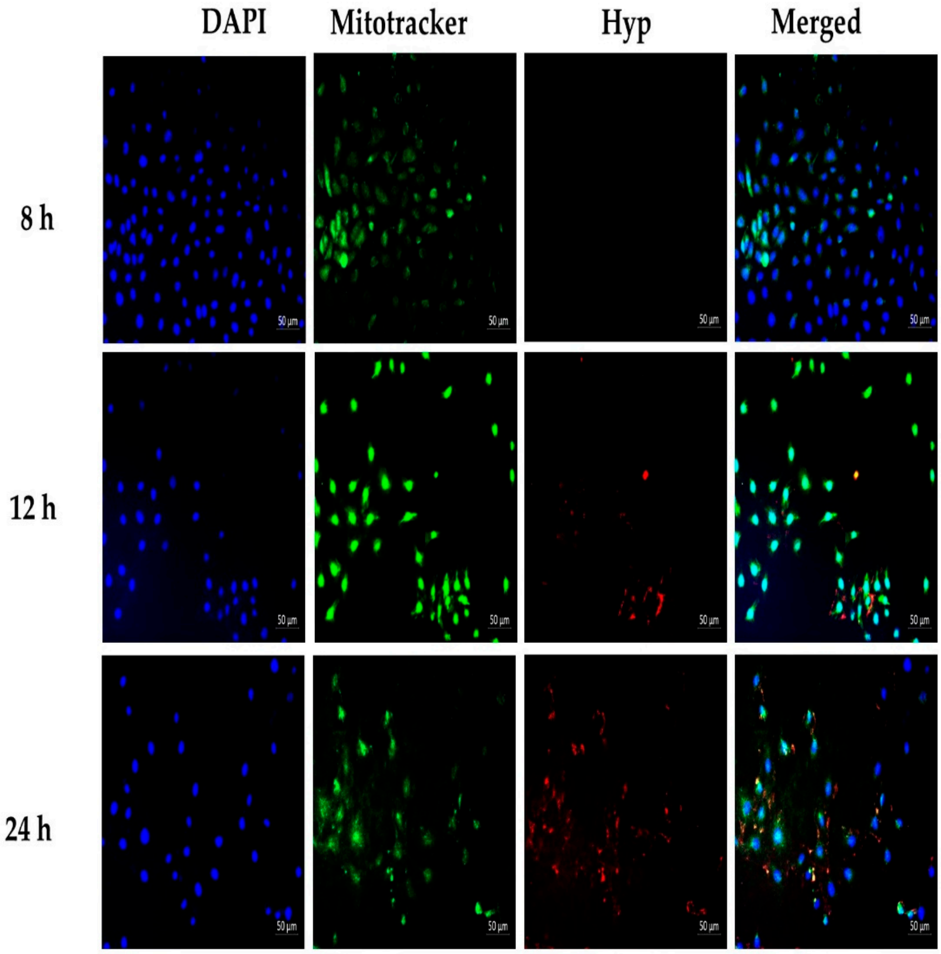

200 x Magnification

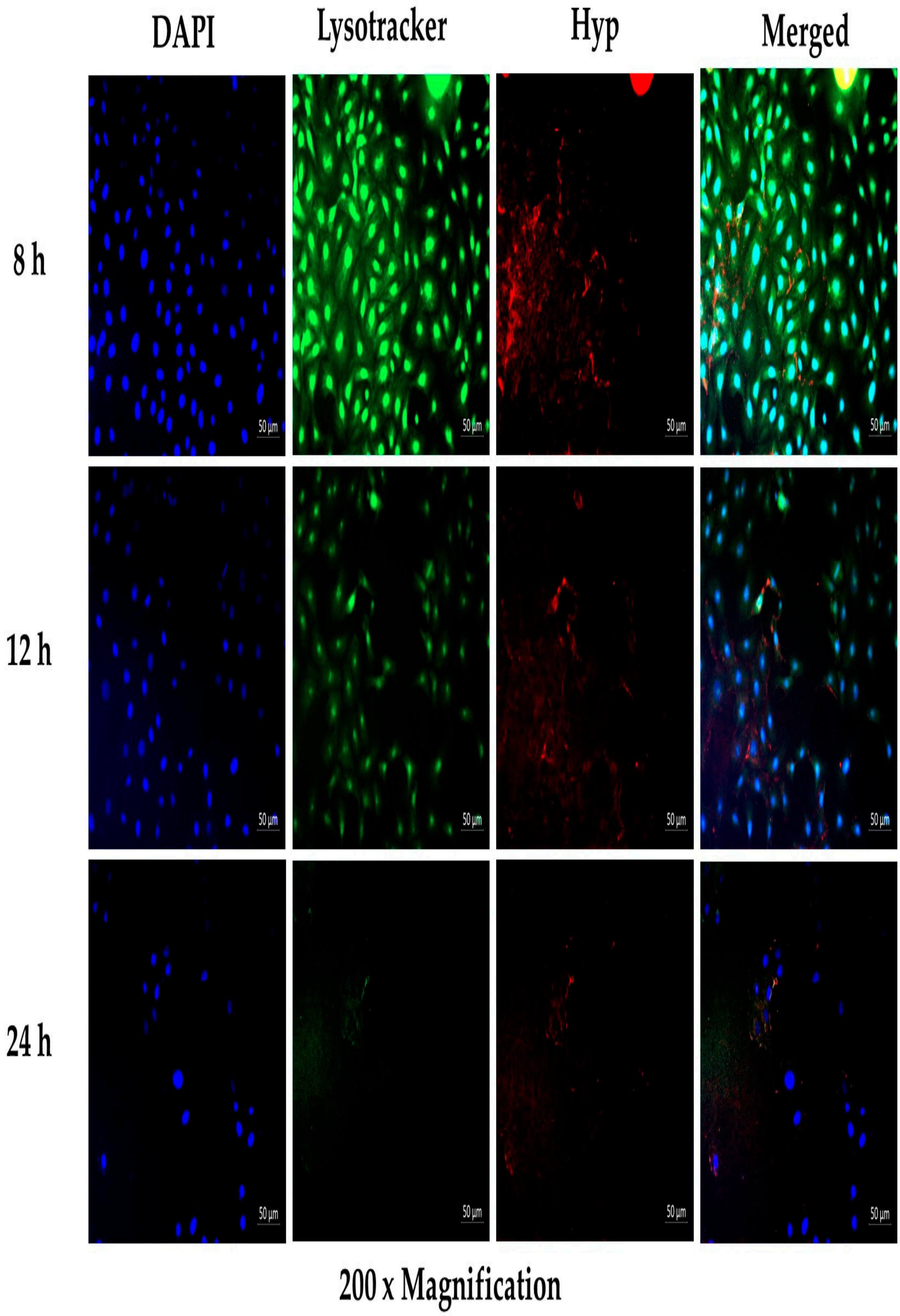
DAPI
Lysotracker
Hyp
Merged
8 h
12 h
24 h
50 µm
200 x Magnification

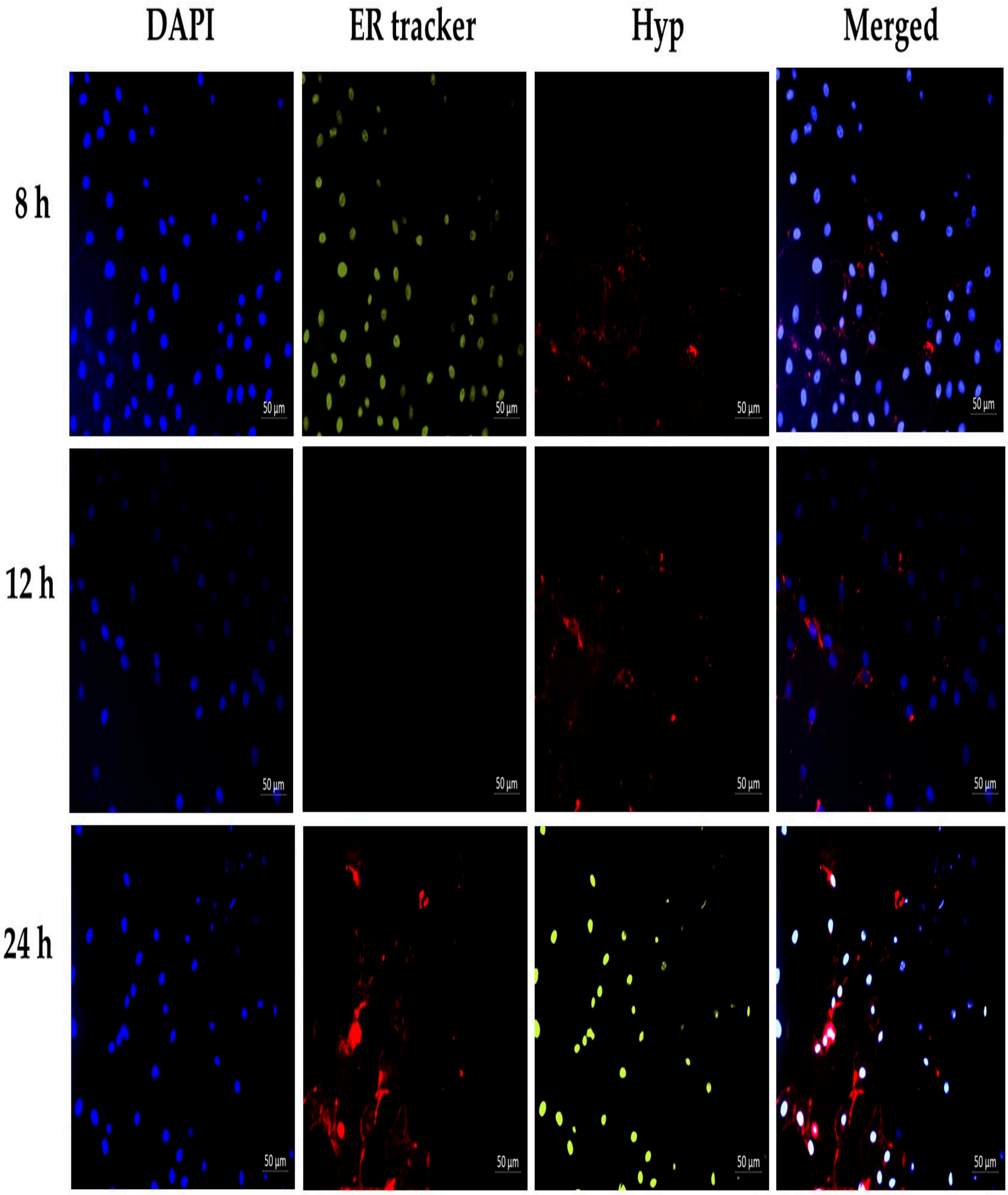

200 x Magnification

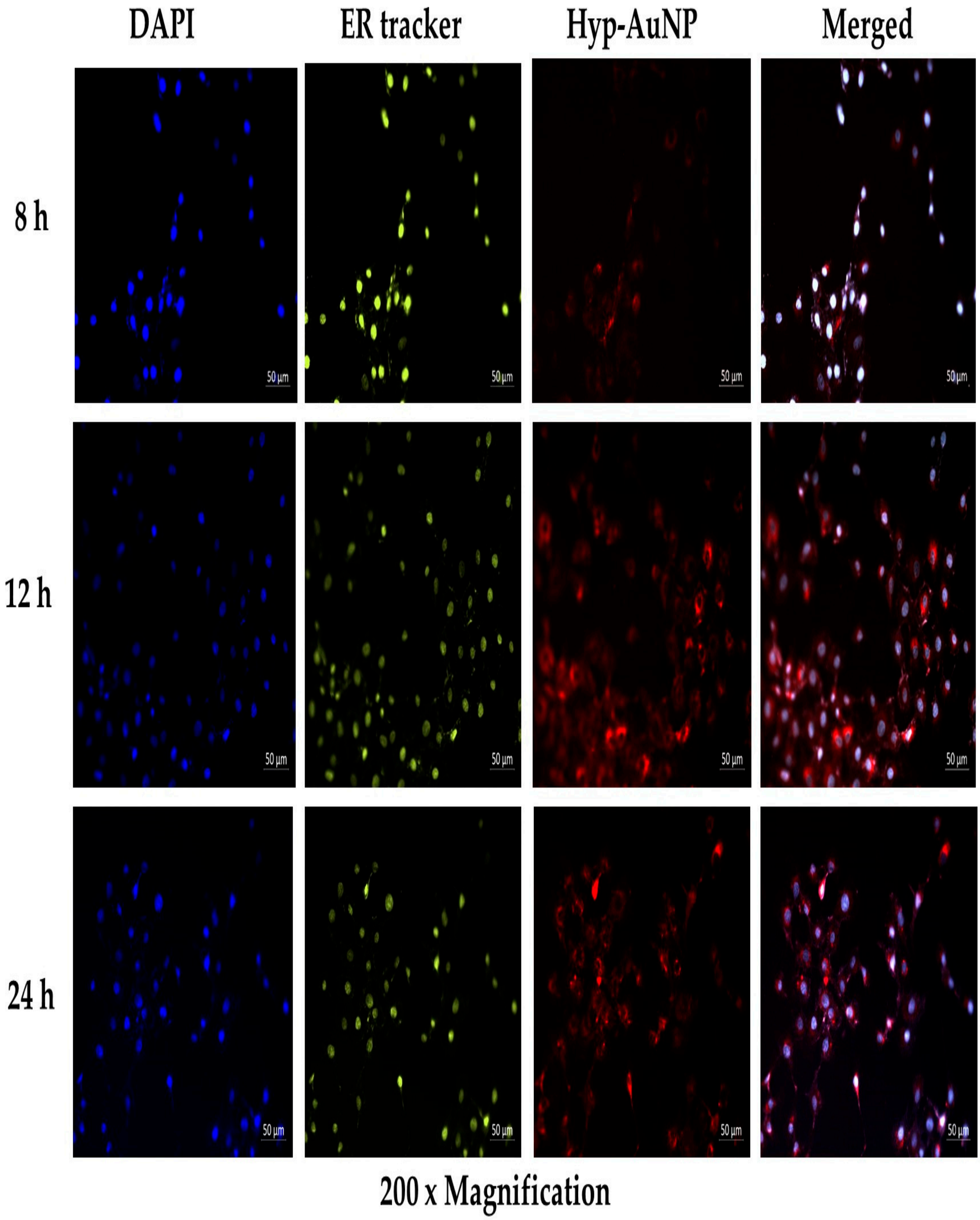
DAPI
ER tracker
Hyp-AuNP
Merged
8 h
12 h
24 h
50 μm
200 x Magnification

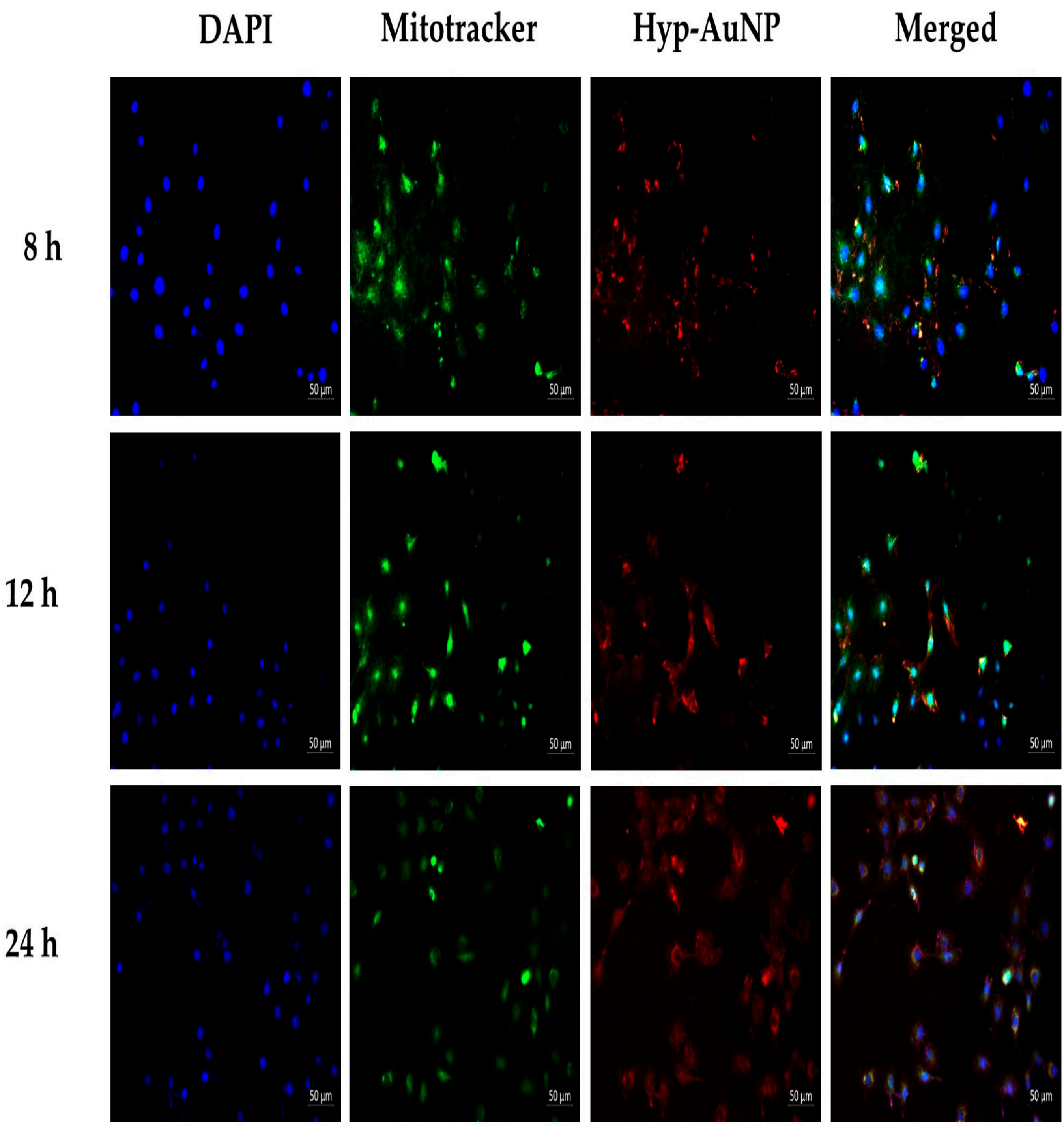

200 x Magnification

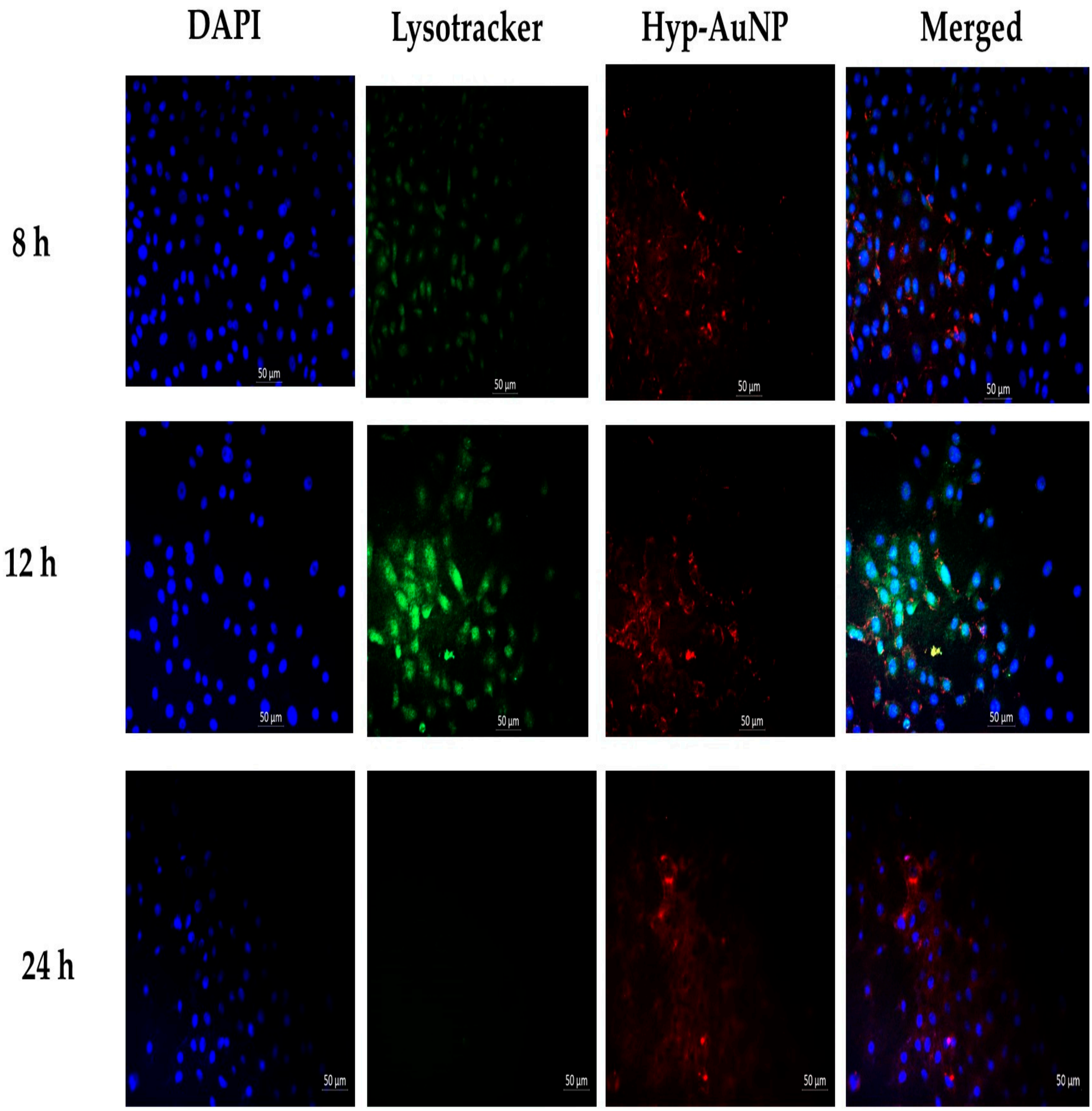

200 x Magnification
